# Sustained success in endoscopic performance demonstrated by the Irish National Endoscopy Quality Improvement Programme

**DOI:** 10.1055/a-2520-9965

**Published:** 2025-02-26

**Authors:** Eoin Keating, Eoin Slattery, Karen Hartery, Glen Doherty, Conor Canavan, Jan Leyden

**Affiliations:** 1 58024 Department of Gastroenterology, St James's Hospital, Dublin, Ireland; 2 8797 School of Medicine, University College Dublin, Dublin, Ireland; 3 58040 Department of Gastroenterology, University Hospital Galway, Galway, Ireland; 4 8799 School of Medicine, University College Galway, Galway, Ireland; 5 8867 National Gastrointestinal Endoscopy Quality Improvement Programme, Royal College of Physicians of Ireland, Dublin, Ireland; 6 8809 School of Medicine, Trinity College Dublin, Dublin, Ireland; 7 58038 Department of Gastroenterology, St Vincent's University Hospital, Dublin, Ireland; 8 8881 Department of Gastroenterology, Mater Misericordiae University Hospital, Dublin, Ireland

**Keywords:** Quality and logistical aspects, Quality management, Performance and complications, Endoscopy Lower GI Tract

## Abstract

**Background and study aims:**

The National Gastrointestinal Endoscopy Quality Improvement (NEQI) Programme captures over 94% of endoscopic activity in the Republic of Ireland (ROI), accounting for > 120,000 colonoscopies per annum. The aim of this study was to assess temporal changes in colonoscopy Key Quality Indicators (KQIs) at a national level over a 5-year period among low-, intermediate-, and high-volume endoscopists.

**Methods:**

A retrospective analysis of all NEQI colonoscopy episodes occurring between 2016 and 2022, collating colonoscopy KQIs (cecal intubation rate [CIR], comfort score [CS], polyp detection rate [PDR] and sedation use). Endoscopists with 5 consecutive years of activity were defined as low, intermediate, or high activity according to annual procedural volumes.

**Results:**

Over 658,000 colonoscopies were completed by 1240 endoscopists. Workload is disproportionate, with 36% of endoscopists completing 66% of national colonoscopy volume. Low-, intermediate-, and high-activity endoscopists all demonstrated sustained improvements in KQI targets over the study period. Comparing experts (≥ 300 colonoscopies/year) vs non-experts, KQI plateaus were demonstrated for PDR at < 150 colonoscopies per year (34.2% vs 29.6%, *P* = 0.002), CS at < 200 procedures per year (97.5% vs 94.9%, *P* < 0.001), and CIR at < 250 colonoscopies per year (94.5% vs 93.4%, *P* = 0.048).

**Conclusions:**

This study represents the first published endoscopist-level NEQI data demonstrating ongoing KQI improvements for endoscopists at all activity levels. Sustaining this improvement and continuing to capture national endoscopic performance will remain a core role of the Irish NEQI program. Workforce imbalances and minimum annual volumes continue to represent challenges for national endoscopy programs.

## Introduction

Quality improvement (QI) has become an essential component of modern medical services. The QI process provides a formal structure for enhancing the quality of care provided, improving both patient safety and workforce skills. Feedback about endoscopy performance data in relation to national and international standards has been shown to be effective in improving endoscopy quality and is endorsed by the Joint Advisory Group on Gastrointestinal Endoscopy and European Society of Gastrointestinal Endoscopy (ESGE) 1 2 3 4 5 .

The ESGE Quality Improvement Committee has identified several key requirements for high-quality gastrointestinal endoscopy reporting systems: automatic data transfer for quality and research purposes; systems should facilitate the inclusion of information on histopathology, patient satisfaction, adverse events, and surveillance recommendations; systems must facilitate easy data retrieval; systems must include data fields key performance indicator data fields as defined by QI committees; and systems must facilitate changes in key quality Indicators (KQIs) and data entry fields as required 6 . Several such national endoscopy QI systems exist, which collate multisite endoscopy performance data from electronic endoscopy reporting systems (ERS), such the National Endoscopy Database (NED) in the UK, the Japan Endoscopy Database (JED) in Japan, and the Dutch Gastrointestinal Endoscopy Audit (DGEA) in the Netherlands 7 8 9 .

In the Republic of Ireland (ROI), the National Gastrointestinal Endoscopy Quality Improvement (NEQI) Programme is one of four National Specialty Quality Improvement programs currently managed by the Royal College of Physicians of Ireland. The goal of NEQI has been to promote a patient-centered QI framework within each Endoscopy Department, routinely reviewing performance and driving improvements in key quality areas against key quality targets. The NEQI Programme has evolved since its inception in 2011, publishing annual national data reports that detail hospital QI data against the key quality targets and national averages across a range of specialty-specific KQIs. These KQIs and their associated targets and recommendations are set out in the National Guidelines for the Implementation of QI Programme in GI Endoscopy 10 . In 2023 the NEQI Programme published its 8th National Data Report 11 .

NEQI data are prospectively collected and analyzed using the National Quality Assurance & Improvement System (NQAIS-Endoscopy). QI data from each hospital ERS are automatically uploaded to NQAIS-Endoscopy and reviewed on a quarterly basis. This identifies participating centers performing to a level consistent with national and international standards, while also recognizing opportunities for underperforming centers to explore local QI to achieve these standards. Individual endoscopists can also track personal KQI rates through their quarterly NQAIS-Endoscopy reports, facilitating self-reflection on performance. However, individual endoscopist reports are not collated in annual reports. NQAIS-Endoscopy reports are also used in tandem with the national training program in order to provide proof of experience and attainment of standards for trainee endoscopists.

A core aim of the NEQI program is to demonstrate quality endoscopy performance to the general population. Public confidence in the diagnostic endoscopy services is enhanced by regular, accessible, and open disclosure of accurate performance information via NEQI’s annual data reports. As of 2022, 94% of Irish hospitals performing endoscopy were enrolled in the NEQI Programme, including all 36 public hospitals 12 .

## Patients and methods

### Study design and aims

We conducted a retrospective analysis of all anonymized NQAIS-Endoscopy reports at each active ROI NEQI participating center, between 2016 and 2022. The primary study aim was to assess temporal trends in KQI performance among high-, intermediate- and low-activity endoscopists tracked by the NEQI Programme. Secondary study aims included the impact of annual colonoscopy volume on KQI attainment.

### Data requests and collection

Formal data requests were submitted to the NEQI for anonymized individual endoscopist NQIAS activity for the period of 2016 to 2022. These data were accessed under General Data Protection Regulation compliance and stored on secure, encrypted hospital servers, with access limited to select study staff (CC, EK, JL, KH, ES, GD). All endoscopists were anonymized prior to data retrieval to ensure individual and site confidentiality.

Annual NQAIS-Endoscopy data were interrogated for each active NEQI endoscopist for the study period. For each anonymized NEQI endoscopist, NQAIS-Endoscopy activity was recorded under the following KQI parameters ( Table 1 ) for each year of the study period (2016–2022). There was no identifiable patient or endoscopist information collected.

**Table:** **Table 1** Anonymized endoscopist NQAIS datapoints.

KQI metric	Scale/unit
Completed colonoscopies	Total colonoscopy volume for calendar year
CIR	Mean CIR for calendar year (%)
CS	Percentage of all colonoscopies scoring ≤ 3 on the Modified Gloucester Comfort Scale
PDR	Mean PDR for calendar year (%)
Midazolam dose (Patient ≥ 70 tears)	Median midazolam dosage (mg) for all patients aged ≥ 70 Years
Fentanyl dose (patient ≥ 70 years)	Median fentanyl dosage (mcg) for all patients aged ≥ 70 years
Midazolam dose (patient < 70 years)	Median midazolam dosage (mg) for all patients aged < 70 years
Fentanyl dose (patient < 70 years)	Median fentanyl dosage (mcg) for all patients aged < 70 years
Sedation reversal episodes	Total volume of colonoscopy procedures requiring sedation reversal
CIR, cecal intubation rate; CS, Comfort Scale; NQAIS, National Quality Assurance & Improvement System; PDR, polyp detection rate.	

NQAIS-Endoscopy format caveats:

1. The current anonymized NQAIS-Endoscopy format is unable to distinguish between Endoscopist 1 and Endoscopist 2 episodes. In the majority of ERS programs, Endoscopist 1 is considered as the primary endoscopist with supervision or assistance provided by Endoscopist 2. This results in duplication of reports for a minority of procedures requiring ≥ 2 endoscopists
2. Endoscopist specialty (e.g. gastroenterologist, surgeon, nurse endoscopist) and training status (e.g. Trainee, fellow, consultant) are not accessible in NQAIS-Endoscopy.
3. Patient demographics (e.g. age, sex) and procedure indications (e.g. colorectal cancer screening (BowelScreen), inflammatory bowel disease surveillance, symptomatic services) are not included in NQAIS-Endoscopy reports.

### Endoscopist population definitions

Annual endoscopist activity was quantified according to the British Society of Gastroenterology (BSG) minimum recommended volumes (≥ 100 colonoscopies) required to maintain competence 13 . Endoscopists exceeding 100 colonoscopies per year were segregated into incremental groups (100–149, 150–199, 200–249, 250–299, ≥ 300) based on annual procedural activity.

#### Exclusion criteria

Endoscopists with less than 100 recorded colonoscopies over the entire study period were excluded to minimize sample bias.

### Consecutive years of activity

Endoscopists with 5 consecutive years of activity between 2018 and 2022 were subanalyzed to assess temporal trends in potential KQI improvement over time. Endoscopists were cohorted according to annual colonoscopy volumes for each year from 2018 to 2022; maximum-activity cohort (5 consecutive years of ≥ 100 colonoscopies, 2018 to 2022), intermediate-activity cohort (minimum of 1 year of ≥ 100 colonoscopies between 2018 and 2022), and low-activity cohort (< 100 colonoscopies in each year 2018 to 2022).

Endoscopists were fixed in these activity cohorts according to their cumulative 5-year activity to ensure no crossover during the study period. KQI results for each activity cohort were compared for each individual year of the study period. KQI results for each activity cohort was compared for each year of the study period.

### Key quality target references

Key quality targets were referenced from the published 2020 NEQI guidelines on endoscopic procedure standards 10 .

### Informed consent

No patient or endoscopist identifiable data are recorded at a national level within the NEQI program. Individual patient or endoscopist informed consent is not required.

### Data analysis

Data analysis and interpretation was completed using IBM SPSS statistics Version 29.0. Categorical variables were described using frequencies and percentages. Mean (± standard deviation) was calculated for continuous data. Comparisons between activity cohorts were completed using independent sample t-tests and one-way analysis of variance tests. Proportions were compared using χ ^2^ (Chi-Square) or Fisher’s exact test. *P* < 0.05 was considered significant.

## Results

### National perspective

Over the 7-year study period (2016–2022), 658,623 colonoscopy episodes were recorded, completed by 1240 individual endoscopists. National colonoscopy volumes increased year-on-year by an average of 10.5% between 2016 and 2019 prior to the interruption of the COVID-19 pandemic and subsequent restricted colonoscopy activity. By 2022, total national colonoscopy volumes had recovered and exceeded 120,000 procedures per year.

National colonoscopy KQIs for CIR, comfort scores, and PDRs exceeded minimum requirements every year and demonstrated an improving trend, despite increasing colonoscopy volumes. Median sedation doses indicate an incremental trend toward lower midazolam doses for all patients. A comprehensive analysis of the national endoscopy trends was published in the 7th NEQI National Data Report in 2022 12 .

### Study endoscopist populations

**Fig. 1:**
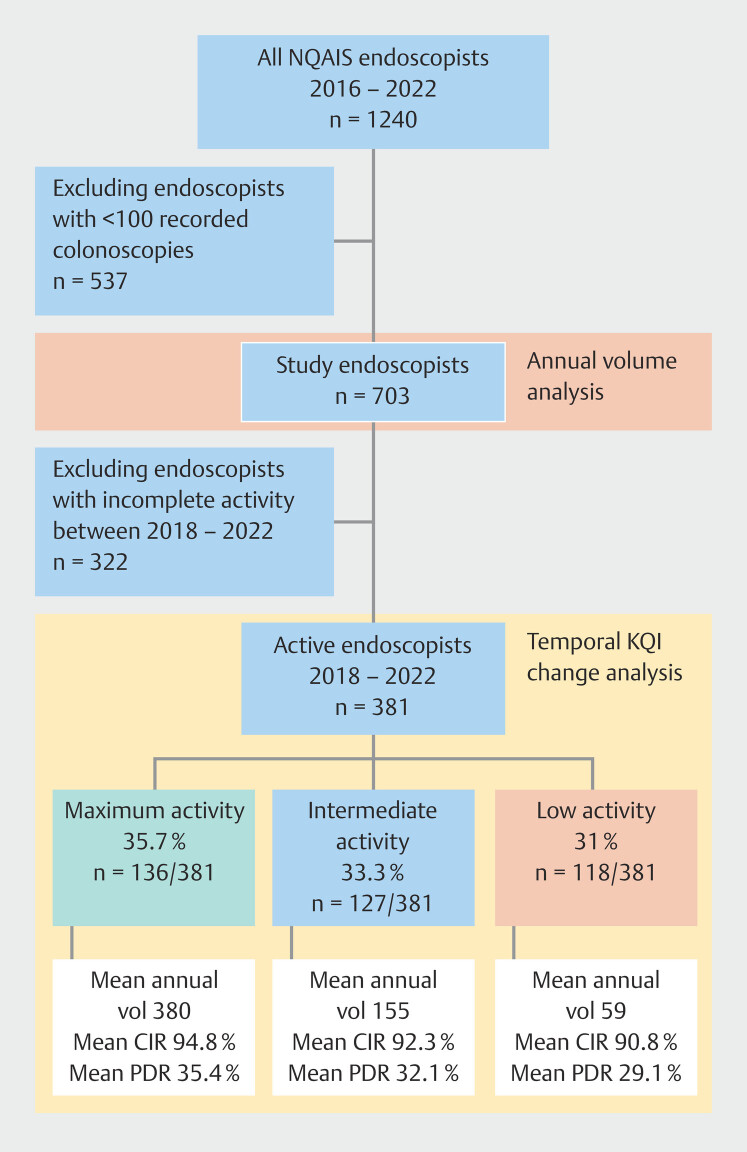
NEQI endoscopist populations.

Data on 1,240 individual endoscopists were captured on NQAIS-Endoscopy reports. Excluding endoscopists with < 100 total NQAIS colonoscopies (n = 537) identified a study population of 703 endoscopists for secondary annual volume analysis. Further analysis was completed on endoscopists with 5 consecutive years of activity between 2018 and 2022 ( Fig. 1 ). These endoscopists accounted for 30.7% (n = 381/1240) of the NQAIS-Endoscopy workforce and amounted to 391,338 recorded colonoscopy procedures.

Maximum-activity endoscopists accounted for 35.7% (n = 136/381) of active endoscopists but completed 66% (n = 258,109/391,338) of colonoscopies completed between 2018 and 2022. Intermediate-activity endoscopists represented 33.3% (n = 127/381) and contributed 25% of national colonoscopy workload (n = 98,924/391,338). Low-activity endoscopists accounted for 31% (n = 118/391) of active endoscopists and the remaining 9% of national colonoscopy volume (n = 34,935/391,338).

**Fig. 2:**
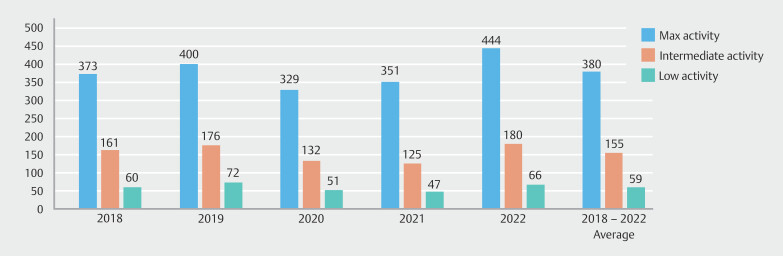
Annual colonoscopy volume vs endoscopist activity 2018–2022.

Maximum-activity endoscopists completed a mean of 380 (± 199) colonoscopies per year between 2018 and 2022. Intermediate endoscopists completed 155 (± 83) colonoscopies per year and low-activity endoscopists performing 59 (± 28) procedures per year during the same time period ( Fig. 2 ). All activity levels experienced reductions in colonoscopy volume due to reductions in endoscopic activity during the COVID-19 pandemic (2020/2021) vs pre-pandemic activity (2018/2019). Low- and intermediate-activity endoscopists experienced more reduction in procedural volume, decreasing to 75% and 76% of pre-pandemic activity respectively. Maximum-activity endoscopists experienced a reduction to 88% of pre-pandemic volumes. In the post-pandemic period (2022), both maximum- and intermediate-activity endoscopists experienced increased procedural volumes, completing 15% and 7% more procedures than in the pre-pandemic period (2018/2019). Low-activity endoscopists returned to baseline pre-pandemic volumes.

### Primary aims

#### Cecal intubation rate

**Fig. 3:**
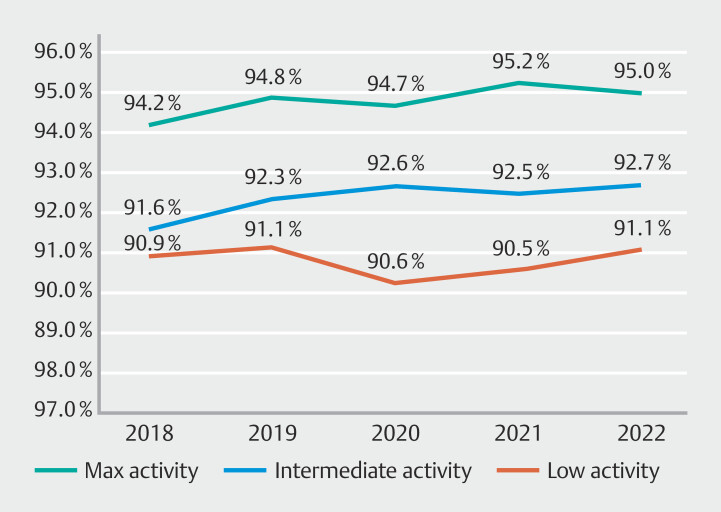
Active endoscopist cecal intubation rate 2018–2022.

Maximum-activity endoscopists significantly outperformed both intermediate- and low-activity endoscopists in CIR KQIs in every year (2018–2022; *P* < 0.001) ( Fig. 3 ). Both maximum- and intermediate-activity endoscopists demonstrated minor CIR KQI improvements over the 5-year study period. Intermediate-activity endoscopists began at equivalent rates as low-activity endoscopists but progressed to significantly outperform them from 2020 to 2022 (2020; 92.6% vs 90.2%, *P* <0.001, 2021; 92.5% vs 90.5%, *P* = 0.011, 2022; 92.7% vs 91.1%, *P* = 0.026).

#### Comfort scores

**Fig. 4:**
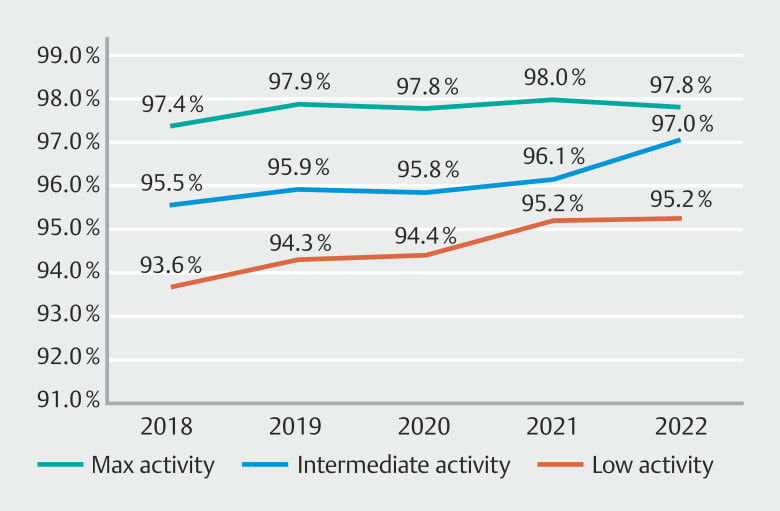
Active endoscopist comfort scores 2018–2022.

Maximum-activity endoscopists significantly outperformed intermediate-activity endoscopists in comfort score KQIs every year (2018–2021; *P* < 0.001, 2022; *P* = 0.032) ( Fig. 4 ). Intermediate-activity endoscopists significantly outperformed low-activity endoscopists every year (2018; *P* = 0.022, 2019; *P* = 0.015, 2020; *P* = 0.019, 2022; *P* = 0.005), with the exception of 2021 (96.1% vs 95.2%, *P* = 0.123) ( Fig. 4 ). All activity levels demonstrated a minor trend toward increased comfort score KQIs over the 5-year period.

#### Polyp detection rate

**Fig. 5:**
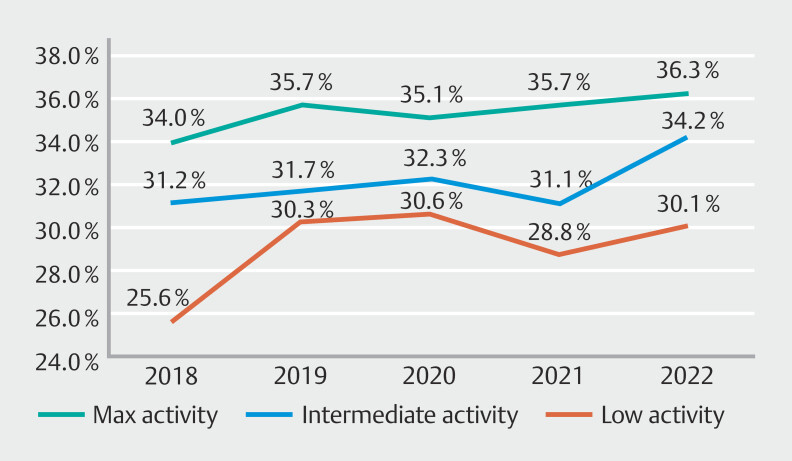
Active endoscopist polyp detection rate 2018–2022.

Polyp detection rates demonstrated the largest KQI improvement over the study period in both intermediate- (31.2% to 34.2%) and low-activity (25.6% to 30.1%) endoscopists ( Fig. 5 ). Maximum-activity endoscopists demonstrated a minor improvement (34% to 36.3%) over the same period.

Maximum-activity endoscopists significantly outperformed low-activity endoscopists in PDR KQI rates in every year (2018–2022; *P* < 0.001, 0.002, 0.015, < 0.001, < 0.001). Maximum-activity endoscopists significantly outperformed intermediate-activity endoscopist in only two of the five study years, 2019 (35.7% vs 31.7%, *P* = 0.009) and 2021 (35.7% vs 31.1%, *P* = 0.007). Intermediate-activity endoscopists similarly outperformed low-activity endoscopists in only two of five study years, 2018 (31.2% vs 25.6%, *P* = 0.001) and 2022 (34.2% vs 30.1%, *P* = 0.017).

#### Sedation patterns

**Midazolam administration**

All endoscopists demonstrated trends toward reduced midazolam doses for both patient age cohorts (< 70 years of age and ≥ 70 years of age) over the study period. Both maximum- and intermediate-activity endoscopists used significantly lower midazolam doses than low-activity endoscopists, in both age cohorts (< 70, ≥ 70), in every study year. There was no significant difference in midazolam dosage between maximum- and intermediate-activity endoscopists for patients aged ≥ 70. At the beginning of the study period, there was a significant difference in dosage for patients aged < 70 (3.5 vs 3.8 mg, *P* = 0.031), but thereafter, there was no significant difference.

**Fentanyl administration**

There was no significant difference in fentanyl dosage for patients aged ≥ 70 years in all activity groups. For patients aged < 70, there was no significant difference between maximum- and intermediate-activity endoscopists (60 vs 62 mcg, *P* = 0.334). Low-activity endoscopists administered significantly higher doses of fentanyl in this age cohort than maximum-activity endoscopists (68 mcg vs 60 mcg, *P* < 0.001) and intermediate-activity endoscopists (68 mcg vs 62 mcg, *P* = 0.013).

**Sedation reversal rates**

Maximum-activity endoscopists had the lowest sedation reversal rates of the activity cohorts at 0.06%. There was no significant difference between maximum- and intermediate-activity endoscopists (0.06% vs 0.09%, *P* = 0.149) or between intermediate- and low-activity endoscopists (0.09% vs 0.12%, *P* = 0.264). Maximum-activity endoscopists had significantly lower reversal rates than low-activity endoscopists (0.06% vs 0.12%, *P* = 0.025).

### Secondary outcomes

#### Annual procedure volume and colonoscopy KQIs (2016–2022)

The study group consisted of 703 endoscopists with a minimum of 100 recorded NQAIS-Endoscopy colonoscopies across the entire study period (2016–2022). This accounted for 644,077 colonoscopy procedures. Endoscopists with an average annual volume < 100 colonoscopies accounted for 45.9% (n = 323/703) of NQAIS endoscopists during the study period. Minimum colonoscopy KQI rates were achieved in all endoscopist volume cohorts 10 . However, KQI rates were significantly higher with annual procedure volumes ≥ 100 colonoscopies vs < 100 colonoscopies including; CIR (93% vs 90.4%, *P* < 0.001), CS (96.2% vs 93.8%, *P* < 0.001) and PDR (32% vs 28.9%, *P* < 0.001).

Increasing average annual procedure volume was significantly correlated with increased KQI rates; CIR (R = 0.303, *P* < 0.001), comfort scores (R = 0.318, *P* < 0.001), and PDR (R = 0.135, *P* < 0.001). Examining endoscopist cohorts with annual volumes exceeding 100 procedures demonstrated a plateau in improvement at different volume thresholds for each colonoscopy KQI. Endoscopists completing ≥ 300 colonoscopies per year achieved the highest CIR (94.5%) and PDR (34.2%) and were used as the expert population. Contrasting experts (≥ 300 colonoscopies/year) vs non-experts, CIR rates continued to significantly improve until a cutoff of < 250 colonoscopies per year (94.5% vs 93.4%, *P* = 0.048). Comfort scores plateaued at a threshold of < 200 procedures per year (97.5% vs 94.9%, *P* < 0.001). PDR reached equivalence at < 150 colonoscopies per year (34.2% vs 29.6%, *P* = 0.002).

## Discussion

### National KQI performance

This study represents the first published endoscopist-level examination of the NEQI Programme. It successfully demonstrates the utility of the NEQI Programme in tracking changes in endoscopist performance over time. By the final year of the study (2022), with 94% of national endoscopic procedures captured, the NEQI Programme demonstrated ongoing improving trends, exceeding the minimum recommended rates for colonoscopy KQI targets. Overall sedation practice continued to shift toward lower sedation doses in all patients with very low sedation reversal rates. A complete analysis of national performance is available in the 8th NEQI National Data Report 11 12 .

### National Endoscopy Quality Programme comparisons

Prospective capture of KQIs such as CIR, PDR and Bowel Preparation scores within NEQI is comparable to the NED, JED, and DGEA 7 9 14 . The NQAIS-Endoscopy format most similarly reflects the comprehensive NED system in the UK, capturing comfort scores, sedation administration, and polyp retrieval rates 7 . Integration of sedation within NQAIS-Endoscopy will facilitate incorporation of the Performance Indicator of Colonic Intubation (PICI) composite validation tool with no additional infrastructure 15 . The recent NED-APRIQOT findings, demonstrating unsustained improvements post-intervention, also support an ongoing feedback process, such as the quarterly schedule currently utilized in the NEQI program 16 . This quarterly schedule is further supplemented by annual reports on each participating NEQI center performance, facilitating peer center comparison.

Because of the ROI’s relatively small population size, the 94% national endoscopic activity capture within NEQI is currently ahead of other national endoscopy databases 12 . However, given the increased focus on QI in all aspects of endoscopy, it is likely that all QI programs will rapidly achieve near total population coverage in the future.

### Endoscopist workload

Endoscopy activity is unevenly distributed among the Irish endoscopy workforce, with 36% of the endoscopists completing 66% of all colonoscopies. This pattern has been recognized in the NED, with gastroenterologists and non-medical endoscopists (NMEs) performing disproportionately more endoscopies than surgical colleagues 17 . In contrast to the NED, with NMEs accounting for 12% of the workforce 17 , Ireland has a smaller proportion of NMEs (< 3%). Applying the NED results to the NEQI dataset suggests that consultant gastroenterologists are likely the largest proportion of the maximum-activity endoscopy group but this requires confirmation. Future iterations of NQAIS-Endoscopy will stratify endoscopists by specialty and training status, providing accurate workload by subspeciality 12 .

Strategies to redistribute endoscopy activity would require significant overhaul of current endoscopy practice, to either encourage increased endoscopic activity among lower-activity endoscopists, or alternatively to curtail their endoscopy activity. Because PDR is integral to future colorectal cancer prevention, the lower KQI rates in this cohort will require intervention if their activity is to be expanded 18 19 20 . This is an essential role of the NEQI Programme, identifying patterns of suboptimal KQI rates versus peers, highlighting needs for further skills training.

In the context of annual increasing demands for more endoscopic procedures, recorded at 10.5% per year, increasing the activity of current low-volume practitioners may be preferable, once improved KQIs can be demonstrated. However, given the rising non-endoscopy-related clinical demands on practitioners, this may not be feasible. This challenge is not unique to an Irish context, with the juxtaposition of increased endoscopy demands versus workforce imbalance evident in the UK 17 , Australia 21 , and the Netherlands 22 .

### KQI trends

Over the study period from 2018 to 2022 inclusive, KQI trends (CIR, CS, and PDR) among all endoscopists demonstrated varying degrees of continuous improvement. The most marked KQI improvements were among intermediate-activity endoscopists, but moderate KQI improvements were also demonstrated in both low- and maximum-activity cohorts. Although the maximum-activity cohort began the study period with KQI rates approaching the aspirational KQI targets, they continued to demonstrate annual improvements. This suggests that even among high-volume practitioners, although there is a slower rate of improvement, there is no ceiling to colonoscopy technical skill.

### Procedure volumes

Although all cohorts demonstrated improvements, the widening gap between KQI rates for low- and intermediate-activity groups supports the current minimum annual volume recommendations (≥ 100 procedures) from the BSG 13 . Increased annual volume is correlated with improved KQI performance, with demonstrable continued improvement in PDR up to 150 annual colonoscopies, comfort scores up to 200 colonoscopies, and CIR up to 250 colonoscopies. Attaining annual volumes of these thresholds is not feasible for some active endoscopists, which factors into minimum-volume suggestions.

### Sedation trends

Overall sedation practice demonstrated a shift towards lower doses in all patient cohorts, even as comfort score KQIs continued to improve. Maximum- and intermediate-activity groups achieved the NEQI KQI midazolam targets for both ≥ 70 and < 70-year-old groups in all study years. Overall sedation reversal rates are reassuringly low, accounting for only 0.1% of all colonoscopy episodes, comparable with 2011 UK data 23 .

### Limitations

This study has several limitations relating to the current NQAIS-Endoscopy format. Procedures with two or more endoscopists may have duplicate recording of procedures, which may affect overall KQI scores, especially among lower-volume practitioners. Endoscopist specialty (e.g. gastroenterologist, surgeon, NME) and training status (e.g. trainee, fellow, consultant) are unknown, which precludes analysis of temporal trends among peer groups. Finally, separation of screening and non-screening colonoscopies is not possible, which may affect PDR reporting, although recent studies have demonstrated no significant difference between these indications 24 . These challenges have been identified in the 8th NEQI Data Report as requirements for future ERS integrations 11 12 .

## Conclusions

This is the first published analysis of the granular, endoscopist-level QI capture of the Irish NEQI Programme. It demonstrates a consistent trend toward improved KQI targets, among low-, intermediate-, and even high-volume endoscopists over the 5-year study period. The NEQI Programme will be integral to monitoring sustained successes in endoscopy performance, highlighting endoscopists for increased training and tracking future workforce changes. Increased annual procedure volumes continue to be associated with improved KQI target scoring but disproportionate endoscopy workforce-to-workload patterns present an ongoing challenge.
